# Gut microbiota modulates osteoclast glutathione synthesis and mitochondrial biogenesis in mice subjected to ovariectomy

**DOI:** 10.1111/cpr.13194

**Published:** 2022-01-26

**Authors:** Yin Yuan, Jing Yang, Aoxiang Zhuge, Lanjuan Li, Shuo Ni

**Affiliations:** ^1^ State Key Laboratory for Diagnosis and Treatment of Infectious Diseases The First Affiliated Hospital, School of Medicine, Zhejiang University Hangzhou 310003 China; ^2^ Department of Orthopedic Surgery and Shanghai Institute of Microsurgery on Extremities Shanghai Jiaotong University Affiliated Sixth People’s Hospital Shanghai 200233 China

**Keywords:** glutathione, Gut microbiota, mitochondrion, osteoclast, reactive oxygen species

## Abstract

**Objectives:**

Osteoporosis is a common bone disease in the elderly mainly regulated by osteoblasts (OBs) and osteoclasts (OCs). The gut microbiota has been recognized as an important factor in many physiological and pathological processes in the host. Thus, we hypothesize that the gut microbiota is necessary for postmenopausal osteoporosis and that germ‐free (GF) mice are protected from osteoporosis.

**Material and Methods:**

Osteoporosis models were established by performing ovariectomy (OVX) in mice. Bone mass was measured by micro‐CT, and gut microbiota were assessed by 16s rDNA sequencing. Reactive oxygen species (ROS) were detected by dihydroethidium (DHE) staining in vivo and 2’,7'‐dichlorodihydrofluorescein diacetate (DCFH‐DA) staining in vitro.

**Results:**

Firmicutes and Bacteroidetes in the intestine are pivotal in OC differentiation, and the Firmicutes/Bacteroidetes ratio (F/B ratio) is a specific indicator of osteoporosis. Furthermore, we found that Firmicutes and Bacteroidetes affect the de novo synthesis of glutathione (GSH) by regulating its key enzyme glutamate–cysteine ligase catalytic subunit (Gclc) and inhibiting mitochondrial biogenesis and ROS accumulation via the cAMP response element‐binding (CREB) pathway. In addition, supplementing OVX mice with the probiotic *Lactobacillus salivarius* LI01 from the Firmicutes phylum prevented osteoporosis.

**Conclusions:**

Our results reveal that GSH plays a vital role in OVX‐induced bone loss, and probiotics that affect GSH metabolism are potential therapeutic targets for overcoming osteoporosis.

## Graphical abstract

Gut microbiota regulates osteoclast differentiation and mitochondrial biogenesis via GSH de novo synthesis.

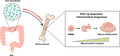

## 1 INTRODUCTION

Osteoporosis is one of the most common bone diseases in the clinic and is mainly caused by a decrease in oestrogen.^1^, ^2^ The disability, mortality and cost of hip and vertebral fractures are substantial in osteoporosis patients.^3^ Therefore, it is a major public health concern to prevent osteoporosis.

Trillions of microbes inhabit the intestine, and these microbes together are referred to as the gut microbiota. The gut microbiota has a profound influence on its host, including regulation of the inflammatory and immune responses and maintenance of homeostasis and other biological processes. Studies have found that colonization with gut microbiota influences bone formation and resorption in germ‐free (GF) mice.^4^ However, the specific mechanism of how gut microbiota influences bone mass remains unclear.

In mammals, bone mass is regulated by osteoblasts (OBs) and osteoclasts (OCs).^5^ OCs play a pivotal role in bone loss induced by decreased oestrogen levels after menopause. The processes of OC differentiation and formation require a large amount of energy. Thus, an increasing number of mitochondria are generated in OCs to maintain the energy requirement in the OC differentiation process.^6^ Peroxisome proliferator‐activated receptor γ coactivator‐1β (PGC‐1β) is a major regulator of mitochondria and can promote the biogenesis of mitochondria in OCs.^7^ PGC‐1β is activated by various signalling pathways, such as the nuclear factor‐κB (NF‐κB), cAMP response element‐binding protein (CREB) and mitogen‐activated protein kinase (MAPK) pathways.^8^, ^9^ In addition to producing ATP, mitochondria produce various metabolic precursors as well as reactive oxygen species (ROS). As an important second messenger, ROS can promote the differentiation and development of OCs.^10^

Glutathione (GSH) is the most important intracellular antioxidant that generates reduction equivalents and inhibits ROS accumulation. The levels of GSH mainly depend on its de novo synthesis. GSH is synthesized in the cytoplasm of almost all cells.^11^ The GSH synthesis process is regulated by its substrates (glutamate, cysteine and glycine) as well as its key enzymes: glutamate–cysteine ligase (GCL) (including the catalytic subunit (Gclc) and the modifier subunit (Gclm) and glutathione synthase (GSS).^12^ Dysregulation of GSH leads to multiple kinds of diseases, including diabetes, liver cirrhosis and drug resistance in cancers.^13^ Thus, it has been increasingly recognized that the regulation of GSH anabolism is a potential target for disease treatments.

In this study, we first proved that the intestinal flora is necessary for the pathological process of bone loss induced by ovariectomy (OVX). Then, by analysing faecal microbes, we found that the percentages of Firmicutes and Bacteroidetes were closely related to OC differentiation. Furthermore, we identified the positive effect of the probiotic *Lactobacillus salivarius* LI01 on the treatment of osteoporosis. *Lactobacillus salivarius* LI01 is a probiotic strain isolated from healthy donors in our laboratory that belongs to the Firmicutes phylum.^14^ Our previous studies showed that LI01 could restore gut microbiome dysbiosis and maintain the integrity of intestinal barriers in response to several kinds of diseases.^15^, ^16^, ^17^ In this work, we demonstrated that LI01 exerts effects by enhancing GSH de novo synthesis, thus inhibiting mitochondrial biogenesis in OCs. Our work provides new insight into the mechanism of bone loss induced by OVX and a new approach for the treatment and prevention of osteoporosis.

## 2 MATERIALS AND METHODS

### 2.1 Animal experiments

All animal experiments followed the guidelines of the Animal Experimental Ethical Inspection of the First Affiliated Hospital, Zhejiang University School of Medicine. Female BALB/c mice were used in our study. Specific pathogen‐free (SPF) mice were purchased from SLACK (Shanghai, China) and raised under conventional (conv) conditions. GF mice were established and raised by our laboratory, and the GF status was regularly verified by assessing faecal 16S rDNA via PCR.

In the first part of our animal experiments, we randomly divided 6‐week‐old SPF and GF mice into 4 groups. Mice in the conv‐sham and GF‐sham groups underwent a sham operation, whereas mice in the conv‐OVX and GF‐OVX groups underwent OVX. Operations were conducted according to our previous work.^18^ All mice were sacrificed after 6 weeks, and samples were collected for further tests.

In the second part of our experiment, we randomly divided 6‐week‐old SPF mice into 3 groups: the sham group underwent sham surgery, and the OVX and LI01 groups underwent OVX. Immediately following surgery, mice in the LI01 group were orally administered with 0.2 ml *Lactobacillus salivarius* LI01 (3 × 10^9^ CFU/ml) everyday while the same volume of normal saline (NS) for mice in the sham and OVX groups. All mice were sacrificed after 6 weeks, and samples were collected.

### 2.2 Probiotic strains and bone marrow‐derived macrophages isolation

*Lactobacillus salivarius* LI01 was isolated from the faeces of healthy donors. LI01 grows anaerobically in Man Rogosa Sharpe (MRS) medium (Oxoid, Thermo Fisher Biochemicals) at 37°C.^14^ Fresh and living LI01 bacteria were used in this study and were washed and re‐suspended in sterile phosphate‐buffered saline (PBS) before use.

Bone marrow‐derived macrophages (BMMs) were isolated from mouse bone marrow in accordance with our previous work.^18^ For cell differentiation, BMMs were cultured in α‐MEM medium containing 20 ng/ml M‐CSF and 50 ng/ml RANKL (R&D). The culture medium was replaced every 2 days until OCs formed.

### 2.3 Intracellular ROS detection

MitoSOX red (Invitrogen, CA, USA) and CellROX green (Invitrogen) were used to detect ROS in mitochondria and cytoplasm, respectively. Briefly, BMMs were cultured with 5 µM MitoSOX and CellROX dyes for 30 mins at 37°C. Then, images were obtained by an LSM T‐PMT confocal microscope (Zeiss).

### 2.4 GSH measurement

GSH levels were assessed by a GSH detection assay kit (Beyotime). Briefly, we collected the supernatant of homogenized tissues to detect the GSH concentrations in them. GSH levels were measured according to the manufacturer's instructions. Absorbance was assessed at 412 nm.

### 2.5 Micro‐CT analysis

Micro‐CT was used to measure femoral bone mass. In brief, samples were scanned at 50 kV and 450 μA by a 0.5‐mm filter and a 9‐μm isotropic voxel. Images were reconstructed with NRecon software and analysed by CT‐Analyzer. Bone volume fraction (BV/TV), trabecular thickness (Tb. Th), trabecular spacing (Tb. Sp) and trabecular number (Tb. N) were calculated by the CTAn program (Bruker microCT).

### 2.6 Glycine, glutamate and cysteine assessment

These three amino acids (AAs) were detected in blood samples collected from the hepatic portal vein. Liquid chromatography–tandem mass spectrometry (LC‐MS/MS) was used in assays.

### 2.7 16s rDNA sequencing

We collected faeces from mice and extracted total DNA with a QIAamp Fast DNA Stool Mini Kit (Qiagen). The V3‐V4 regions were amplified and then sequenced in Oebiotech™. Sequence data have been uploaded to the NCBI Sequence Read Archive.

### 2.8 Western blotting

Proteins were extracted by RIPA lysis buffer (Beyotime) containing protease inhibitors and phosphatase inhibitors. SDS‐PAGE was used to separate the proteins. Then, proteins were transferred to PVDF membranes and probed with specific primary antibodies and secondary antibodies. Primary antibodies were purchased from CST (Beverly) and Boster Biological Technology (Wuhan, China). Bands were visualized by an enhanced chemiluminescence detection kit (Beyotime) and were quantified by ImageJ software.

### 2.9 RNA extraction and qRT‐PCR

RNA was extracted with RNeasy Mini Kits (Qiagen) and reverse‐transcribed to cDNA with PrimeScript™ RT reagent kits (Takara). The cDNA abundance was assessed with a VII A7 real‐time PCR system (Applied Biosystems). Primers are listed in Table S1.

### 2.10 Femur histomorphometry

For tartrate‐resistant acid phosphatase (TRAP) staining, paraffin‐embedded femurs were stained with TRAP and haematoxylin according to the protocol of the TRAP staining kit (Sigma Aldrich). For dihydroethidium (DHE) staining, frozen femurs were stained with DHE in accordance with the procedures reported.^19^

### 2.11 Statistical analysis

SPSS software and GraphPad Prism 7.0 were used to analyse the data. Student's t‐test and ANOVA were used to calculate the differences among groups. *p* < 0.05 was considered as significance.

## 3 RESULTS

### 3.1 GF mice are protected from OC differentiation induced by OVX

**FIGURE 1:**
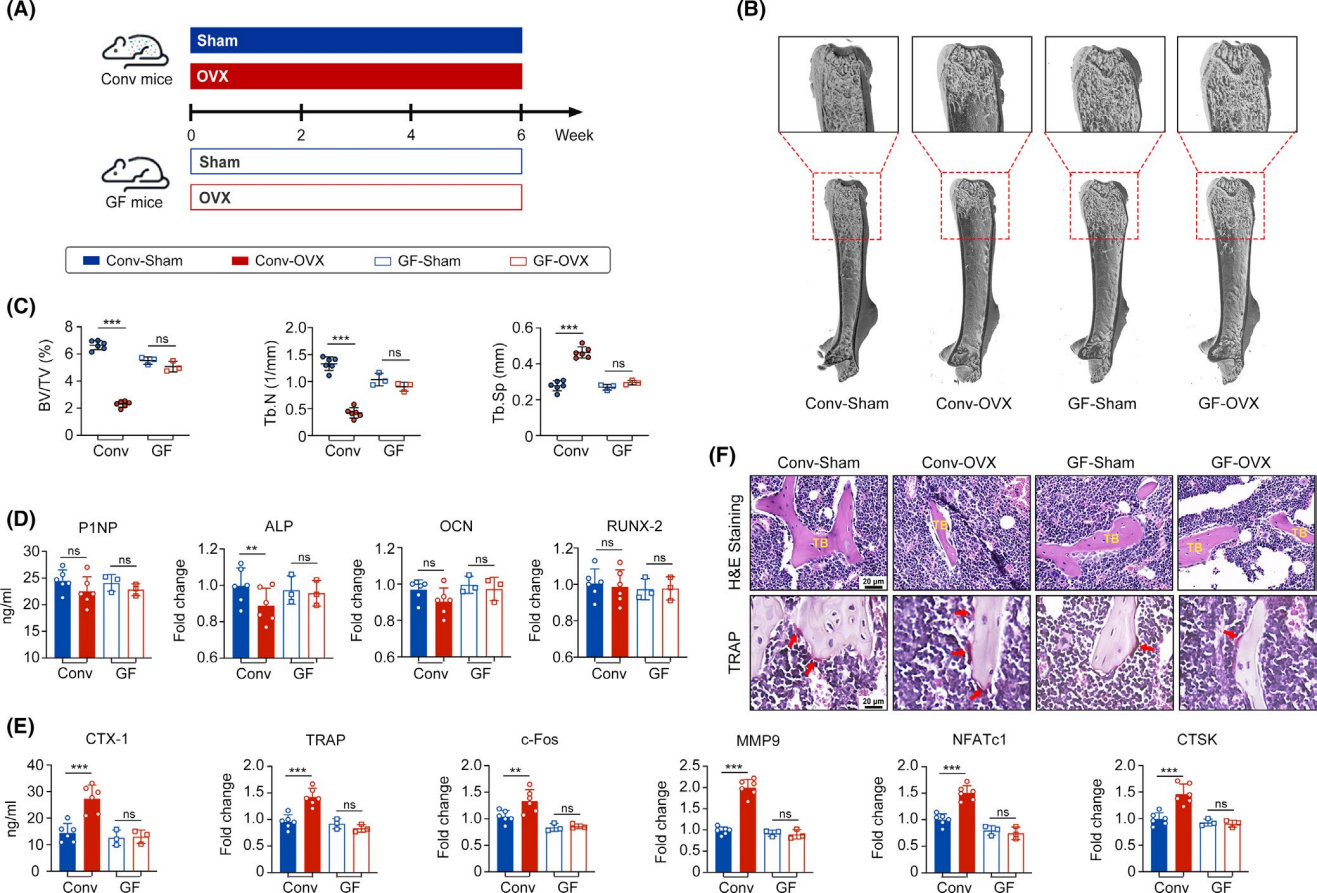
Germ‐free (GF) mice are protected from OVX‐induced bone loss and osteoclasts differentiation. (A) Schematic diagram of animal experiment. Conventional (conv) mice were kept in specific pathogen‐free (SPF) condition, and germ‐free (GF) mice were maintained in GF condition before sacrificed. (B‐C) Representative images of micro‐CT and quantification of bone volume/tissue volume (BV/TV), trabecular number (Tb.N) and trabecular separation (Tb. Sp). (*n* = 6 mice in conv condition and *n* = 3 mice in GF condition). (D) qRT‐PCR analysis of osteoblastic biomarker genes P1NP, ALP, OCN and Runx‐2. (E) qRT‐PCR analysis of osteoclastic biomarker genes including CTX‐1, TRAP, c‐FOS, MMP9, NFATc1 and CTSK. F H&E and TRAP staining of femur tissues. Osteoclasts are indicated by red arrows; TB, trabecular bone. (ns, no significance; **p < 0.01; ***p < 0.001)

To investigate whether bone mass and structure were regulated by gut microbes, two groups of female BALB/c mice were fed under conventional (conv) and GF conditions. Micro‐CT scanning was used to measure bone mass. No difference in distal femur length and bone mass was detected in 6‐week‐old conv mice and GF mice (Figure S1A‐D), suggesting that the presence of commensal microbes did not affect bone mass in the young and adolescent mice. Then, we established mouse models of OVX‐induced osteoporosis (Figure 1A). Compared with the conv‐sham group, the conv‐OVX group presented significant bone loss reflected as decreases in the bone volume fraction (BV/TV) and trabecular number (Tb.N) and an increase in trabecular spacing (Tb. Sp), whereas no difference was observed between the GF‐sham and GF‐OVX groups (Figure 1B‐C). Trabecular bone mass reflects the balance between bone formation (i.e. OBs) and resorption (i.e. OCs).^5^ Thus, we measured osteoblastic and osteoclastic biomarkers by qRT‐PCR. The levels of the osteoblastic biomarkers (P1NP, ALP, OCN and Runx‐2) remained almost unchanged in the OVX groups under conv and GF conditions (Figure 1D). However, the expression of osteoclastic biomarkers, including CTX‐1, TRAP, c‐FOS, MMP9, NFATc1 and CTSK was significantly increased in conv‐OVX mice but not in GF‐OVX mice (Figure 1E). H&E and TRAP staining was also used to histomorphometrically measure bone mass and OCs in femur tissues, and similar results were observed (Figure 1F). Thus, we speculate that gut microbes are necessary for OVX‐induced OC differentiation and that GF mice are protected from this differentiation.

### 3.2 Gut microbes are necessary for OVX‐induced ROS accumulation in bone marrow

**FIGURE 2:**
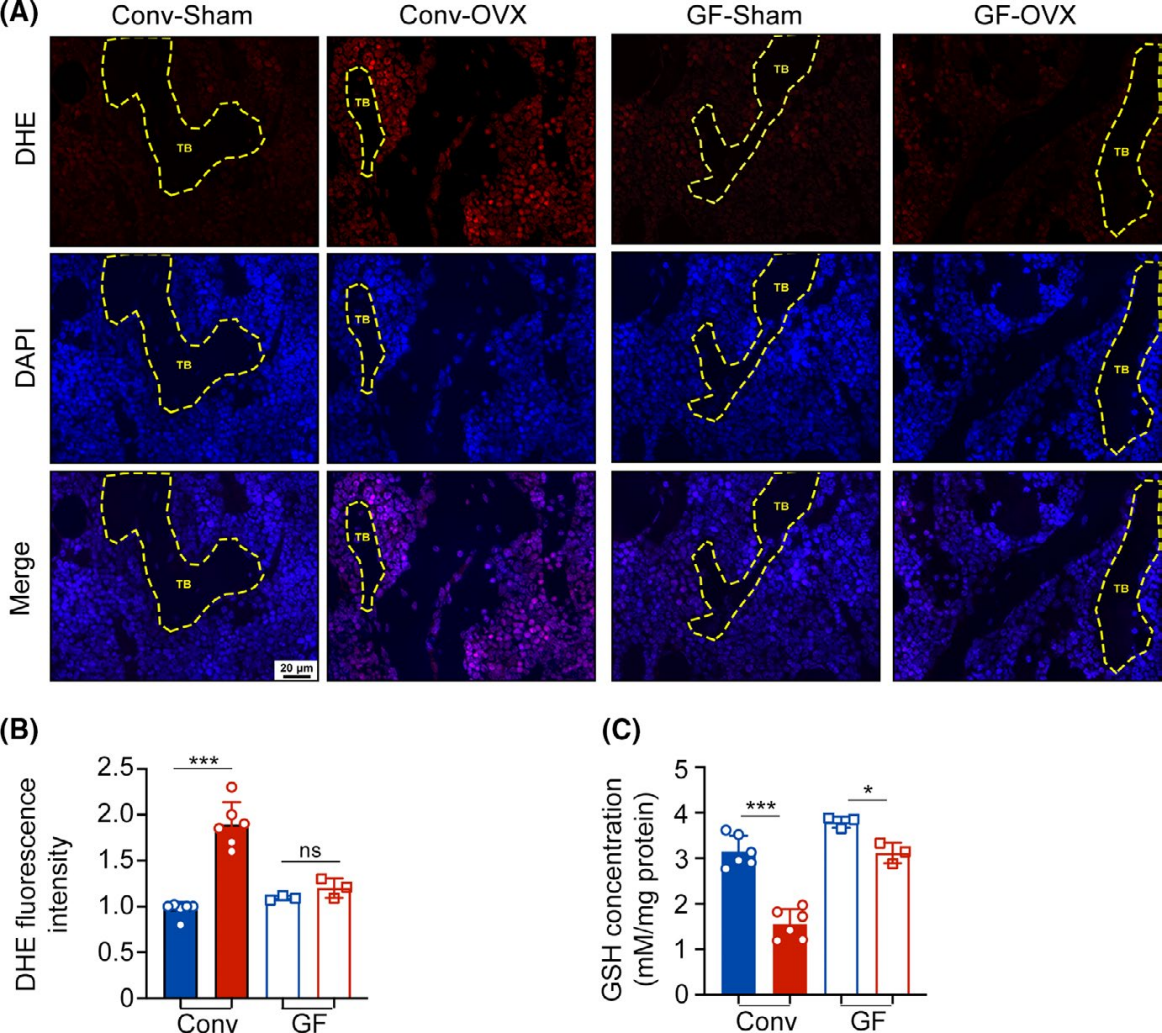
Gut microbes are necessary to OVX‐induced ROS accumulation in bone marrow. (A‐B) Images and quantification of femoral dihydroethidium (DHE) staining. (C) Glutathione (GSH) concentrations in bone marrow tissues. Results are shown as mean ± SD. (ns, no significance; **p < 0.01; ***p < 0.001)

ROS levels in bone marrow are crucial in OC differentiation.^20^, ^21^ In our work, significant accumulation of ROS was detected in the conv‐OVX group, but no differences were observed between the GF groups (Figure 2A‐B). GSH is one of the most important intracellular ROS scavengers^11^; thus, we detected GSH levels in bone marrow. Surprisingly, the average GSH levels in conv‐OVX mice were significantly lower than those in conv‐sham mice, but no differences were observed in the GF groups (Figure 2C). Thus, we speculate that gut microbes participate in OC differentiation by influencing ROS and its scavenger GSH.

### 3.3 OVX induces gut microecological dysregulation in mice

**FIGURE 3:**
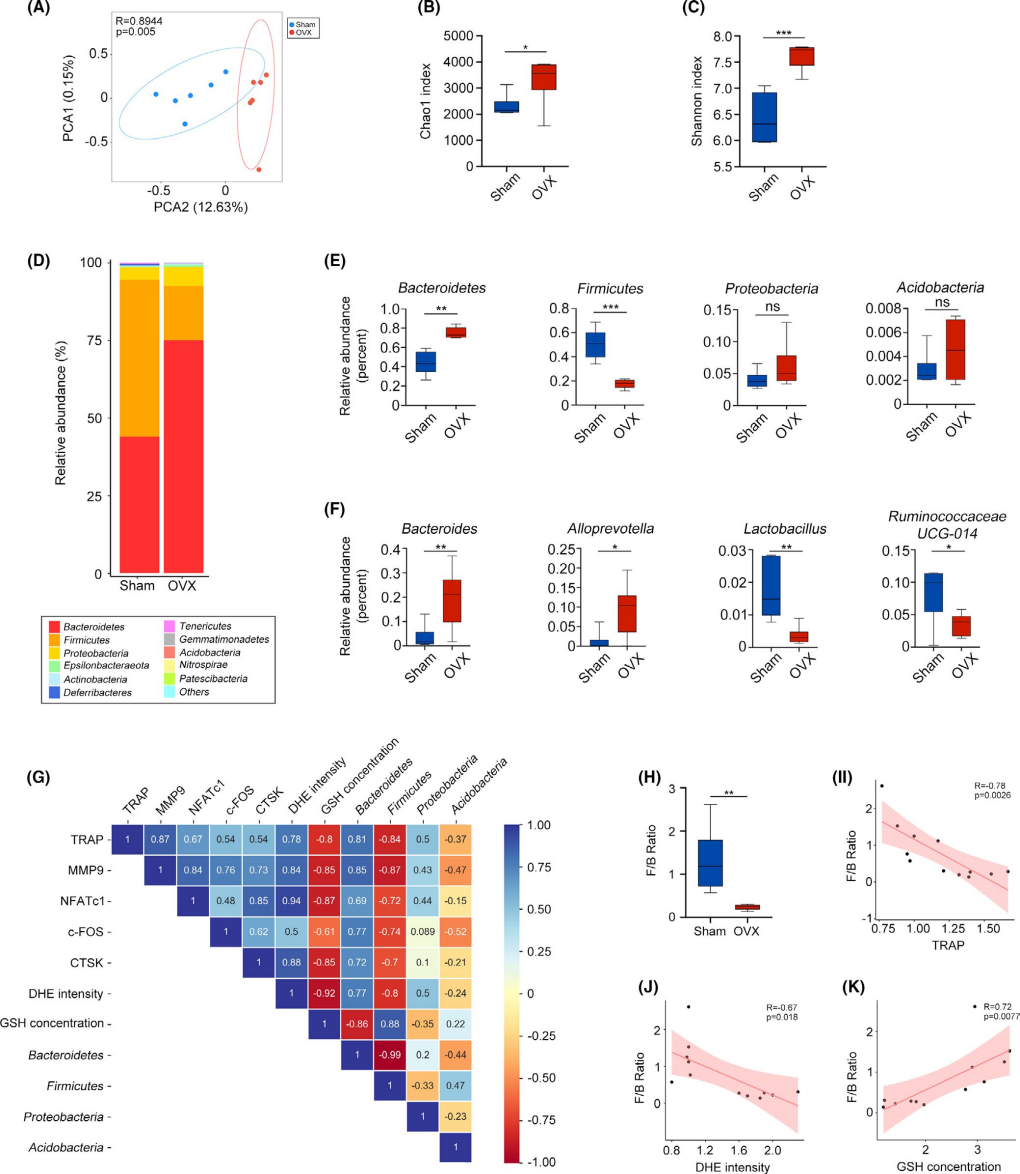
Gut microecology alters in OVX mice. (A) Beta diversity comparison basing on PCA analysis. (B) Community richness evaluated by Chao1 index. (C) Alpha diversity represented by Shannon index. (D‐F) Relative abundance of microbial taxa at the phylum level (D, E) and the genus level (F). G Pearson correlation analysis of microbial taxa, osteoclastic genes, DHE intensity and GSH concentration. (H) Firmicutes/Bacteroidetes (F/B) ratios in the sham group and the OVX group. Results are shown as mean ± SD. I‐K Pearson correlation analysis of F/B ratio, TRAP, DHE intensity and GSH concentration. (ns, no significance; *p < 0.05; **p < 0.01; ***p < 0.001)

To explore potential correlations between gut microbiota and OC differentiation, we collected faeces from mice and performed 16S rRNA sequencing analysis. Significant differences in microbial diversity were found between the sham and OVX groups (Figure 3A‐C). At the phylum level, Firmicutes and Bacteroidetes were the two dominant florae in both groups. Interestingly, compared with the sham group, the OVX group showed a significant decrease in the relative abundance of Firmicutes and an increase in Bacteroidetes abundance (Figure 3D‐E). At the genus level, increased abundances of *Bacteroides* and *Alloprevotella* from the Bacteroidetes phylum were observed in the OVX group, while the abundances of *Lactobacillus* and *Ruminococcaceae* UCG‐014 from the Firmicutes phylum were decreased (Figure 3F). According to these results, we speculate that the Bacteroidetes and Firmicutes abundances are related to osteoporosis and that they are potential indicators of bone mass.

### 3.4 The F/B ratio is a specific indicator of OC differentiation, ROS levels and GSH concentrations in mice

Next, Pearson correlation analysis was performed to further explore whether OC differentiation was affected by specific microbial species. We found that the levels of OC‐related genes and ROS levels were positively correlated with the abundance of Bacteroidetes and negatively correlated with the abundance of Firmicutes, while GSH levels showed the opposite correlation (Figure 3G). More accurately, we calculated the F/B ratio and surprisingly found that the average F/B ratio in the OVX group was significantly reduced (Figure 3H) and was negatively correlated with TRAP and ROS levels but positively correlated with GSH concentration (Figure 3I‐K). Therefore, we speculate that Firmicutes and Bacteroidetes play pivotal roles in OVX‐induced OC differentiation and ROS and GSH concentrations and that the F/B ratio is a specific indicator of these processes.

### 3.5 Supplementation with *Lactobacillus salivarius* LI01 increases the F/B ratio and prevents OVX‐induced OC differentiation by increasing GSH levels

**FIGURE 4:**
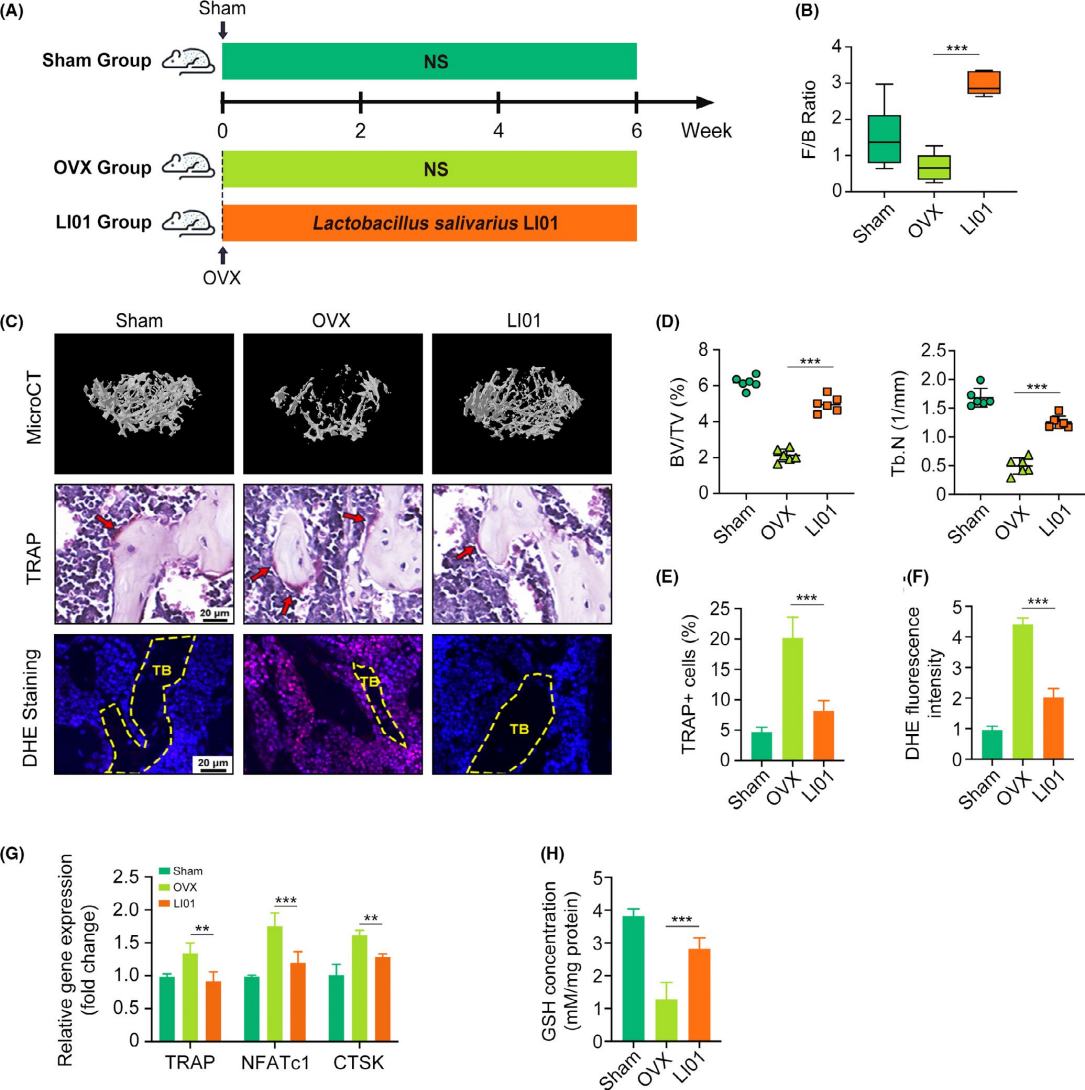
*Lactobacillus salivarius* LI01 prevents OVX‐induced OCs differentiation and ROS accumulation. (A) Figure protocol of animal experiment. Mice in the LI01 group are orally given 0.2 ml *Lactobacillus salivarius* LI01 (3 × 109 CFU/ml) every day. (B) Firmicutes/Bacteroidetes (F/B) ratios in three groups. (C‐E) Images and quantifications of femoral micro‐CT, H&E and TRAP staining. (G) Osteoclastic biomarker genes TRAP, NFATc1 and CTSK assessed by qRT‐PCR. (H) Glutathione (GSH) concentrations in bone marrow tissues. (**p < 0.01; ***p < 0.001)

Next, we supplemented OVX mice with *Lactobacillus salivarius* LI01 (a probiotic from the Firmicutes phylum) (Figure 4A). No change was found in the total body weight or uterus weight in mice between the OVX and LI01 groups (Figure S2A‐B). Several studies have reported that OVX mice tended to gain more weights than sham group.^22^ However, our results were inconsistent with these studies. This may be due to dietary and environmental factors, and we will explore it in further studies. The faecal F/B ratio increased in mice supplemented with LI01, suggesting that this probiotic could firmly colonize in the intestinal tract and could restore microecological homeostasis (Figure 4B). Meanwhile, bone mass and GSH concentration increased while OC‐related gene expression and ROS levels decreased in mice administered LI01 (Figure 4C‐H). Thus, we concluded that the probiotic LI01 can restore gut microecological homeostasis, increase the F/B ratio and prevent OVX‐induced OC differentiation by upregulating GSH levels and inhibiting ROS accumulation.

### 3.6 *Lactobacillus salivarius* LI01 enhances GSH de novo synthesis in mice

**FIGURE 5:**
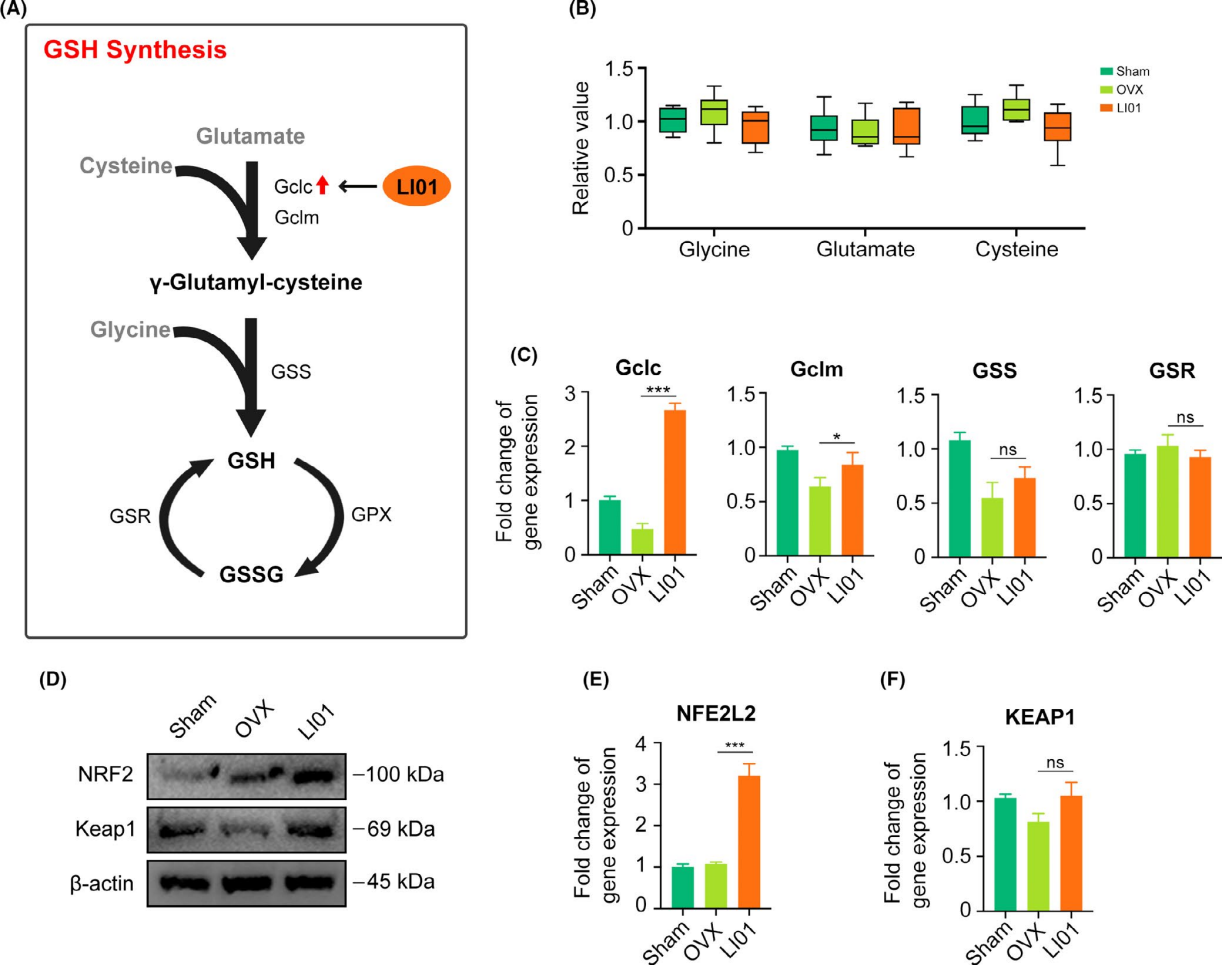
*Lactobacillus salivarius* LI01 enhances GSH de novo synthesis by increasing NRF2. (A) Substances and metabolic enzymes involved in GSH de novo synthesis. Red arrow indicates an increase in Gclc in LI01 group. (B) Relative level of glycine, glutamate and cysteine. (C) qRT‐PCR analysis of Gclc, Gclm, GSS and GSR genes. (D) Western blotting bands of NRF2 and Keap1 in bone marrow tissues. (E‐F) qRT‐PCR analysis of NRF2 gene (NFE2L2) and Keap1 gene. (ns, no significance; *p < 0.05; ***p < 0.001)

In vivo, GSH is synthesized from three substrates (glycine, glutamate and cysteine) and catalysed by key enzymes, including GCL (Gclc and Gclm subunits), GSS and GSR (Figure 5A). Several studies have reported that gut microbes can influence the absorption and metabolism of AAs in their host. Hence, we examined whether LI01 increased GSH synthesis by affecting AA metabolism in the host, but no difference was observed in the levels of all three substrates (Figure 5B). Next, we measured the gene expression of those key enzymes. Surprisingly, the qPCR results showed that LI01 administration could upregulate the gene expression of Gclc and Gclm with little effect on GSS and GSR (Figure 5C). These results suggested that LI01 maintains REDOX homeostasis by activating the key GSH synthesis enzyme GCL. Numerous studies have demonstrated that Gclc and Gclm are regulated by a variety of signalling pathways and transcription factors.^12^, ^23^ Thus, we detected several pathway‐related inflammatory cytokines and the major transcription factor NRF2. The protein levels of NRF2 increased in the OVX and LI01 groups, while Keap1 protein level decreased only in the OVX group. However, the mRNA expression of NRF2 (also named NFE2L2) increased with the treatment of LI01, whereas Keap1 mRNA level remained unchanged (Figure 5D‐E). Given that the combination of NRF2 and Keap1 restrains NRF2 degradation, we speculate that LI01 transcriptionally activated Gclc by increasing NRF2 gene expression and lessening its degradation. However, administration of LI01 inhibited the expression of cytokines including TNF‐α, IL‐6 and TGF‐β in both colon and bone marrow (Figure S3A‐B). Several published studies revealed that high expression of cytokines could activate GCL enzymes.^24^ These studies were opposite to our results, and thus we thought that cytokines were not a potential mechanism to Gclc in response to LI01, and we will investigate this issue in our future studies.

### 3.7 GSH suppresses mitochondrial biogenesis by inhibiting CREB activation in vitro

**FIGURE 6:**
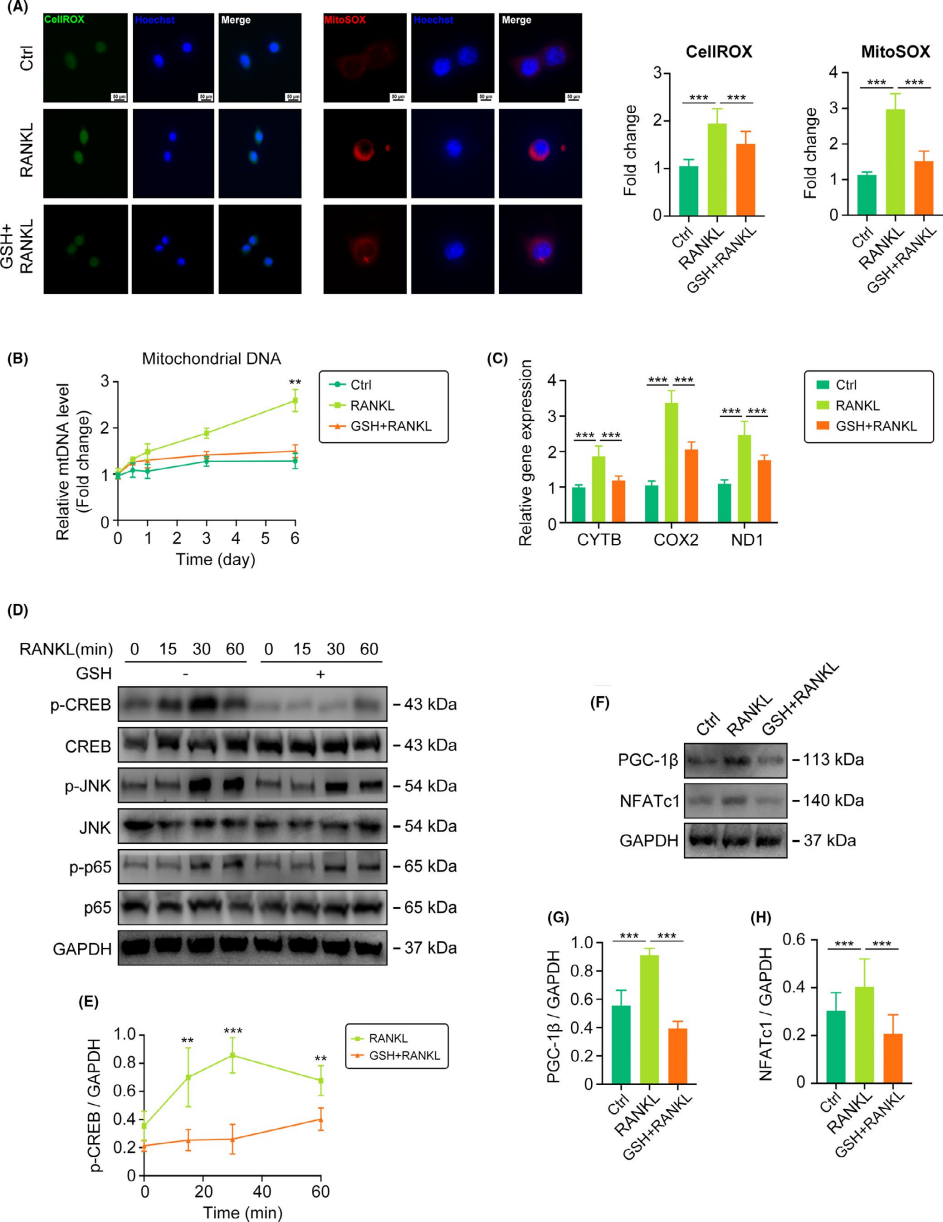
GSH suppresses mitochondria biogenesis by inhibiting CREB activation in vitro. (A) Cytoplasmic ROS detected by CellROX (green) and mitochondrial ROS by MitoSox (red) in BMMs. (B) Quantification of mitochondrial DNA (mtDNA) in 0–6 days. (C) qRT‐PCR analysis of respiratory chain genes CYTB, COX2 and ND1. (D‐E) Total and phosphorylated protein levels of CREB, JNK, p65 measured by Western blotting in BMMs. (F‐H) Western blotting bands and quantification of PGC‐1β, NFATc1. (**p < 0.01; ***p < 0.001)

To further elucidate the mechanisms involved in this phenomenon, we extracted BMMs from mice and stimulated them with RANKL. By using CellROX and MitoSOX dyes to quantify and localize cytoplasmic and mitochondrial ROS, respectively, in BMMs, we found that both types of ROS could be eliminated by GSH, but the decrease in mitochondrial ROS was more pronounced (Figure 6A). Mitochondria are unique double‐membrane organelles that produce energy and ROS.^25^ The level of mitochondrial DNA (mtDNA) in BMMs gradually increased with RANKL stimulation, and the expression of respiratory chain genes CYTB, COX2 and ND1 increased, indicating that the newly formed mitochondria were in action. These changes were all mitigated by GSH (Figure 6B‐C). Next, we tested several signalling pathways related to mitochondrial biogenesis and found that only the CREB pathway was inactivated by GSH (Figure 6D‐E). PGC‐1β is reported to be a pivotal gene in mitochondrial biogenesis in OCs and can be activated by the CREB pathway.^8^ Our work demonstrated that GSH reduced PGC‐1β levels and inhibited mitochondrial biogenesis by inactivating the CREB signalling pathway. In addition, activated CREB could also transcriptionally activate the expression of the OC‐related gene NFATc1 and jointly affect OC differentiation (Figure 6F‐H).

## 4 DISCUSSION

Trillions of bacteria colonize the human intestine and participate in many kinds of physiological processes.^26^, ^27^ Previous studies have shown that gut microbes can affect the host's bone mass^28^, ^29^; however, the correlation between specific kinds of microbes and bone mass has not been clearly clarified. Through 16S rRNA gene sequencing analysis, we found that the abundances of Firmicutes and Bacteroides changed significantly in mice subjected to an osteoporosis model. More precisely, a negative correlation between faecal F/B ratios and OC differentiation was found by bioinformatics analysis.

Firmicutes and Bacteroides are the two main components of mammalian gut flora, and they play important roles in maintaining gut microecological homeostasis.^30^, ^31^ Several studies have reported that alterations in Firmicutes and Bacteroides abundances result in a variety of diseases, and the potential mechanisms include excessive immune activation or the production of beneficial metabolites such as SCFAs.^32^, ^33^, ^34^ Our experiments demonstrated that ROS are the relevant mechanism, and oral administration of the probiotic *Lactobacillus salivarius* LI01 (a probiotic from Firmicutes phylum) rescued the bone resorption after OVX. However, the structure of gut microbes varies from person to person and is affected by many factors, among which dietary type and race were the most important. Those factors in mice experiments are easy to control, whereas human researches are not. Therefore, further studies are still needed.

Next, we assessed the level of GSH, the most important ROS scavenger. The synthesis of GSH is regulated by the contents of its substrates and the activities of its key enzymes. Several studies have reported that the intestinal flora can regulate AA metabolism in their hosts. However, we found that LI01 did not increase GSH by regulating the anabolism of AA but rather by increasing Gclc expression, a key enzyme subunit involved in GSH de novo synthesis. Further exploration of how LI01 regulates Gclc was similar to the study reported by Kang et al.^23^ Kang and colleagues stated that NRF2 activation could promote Gclc gene expression. In our work, administration of LI01 enhanced the mRNA expression of NRF2 and thus transcriptionally activated Gclc. However, given that Gclc could be regulated by several signalling pathways, we measured the levels of some related inflammatory cytokines, but conflicting results were observed. Therefore, we speculated that cytokines were not a potential mechanism to Gclc in response to LI01. We will investigate this issue in further studies.

The number of mitochondria gradually increases following the process of OC differentiation.^25^ RANKL stimulation activates CREB by causing calcium oscillation in BMMs and enhances the gene expression of PGC‐1β, thus promoting mitochondrial biogenesis in BMMs.^35^ Apart from producing ATP for energy, mitochondria produce a large amount of oxygen radicals, resulting in ROS accumulation and thus promoting OC differentiation. We found that GSH not only directly eliminated ROS but also reduced ROS production by inhibiting mitochondrial biogenesis. However, a study conducted by Wilson et al. clarified that the biogenesis of mitochondria in BMMs was regulated in either a PGC1β‐dependent or PGC1β‐independent pathway and that PGC‐1β could regulate the differentiation of OCs by affecting other factors.^36^ Further studies are still needed to confirm these data.

In conclusion, in this study, we showed that gut microbes are necessary for OVX‐induced OC differentiation in mice, which is accompanied by ROS accumulation in bone marrow and alterations in GSH synthesis. By analysing the gut microbiota of mice, we concluded that the faecal F/B ratio can serve as a specific index to evaluate the degree of OC differentiation and GSH synthesis. Supplementing OVX mice with *Lactobacillus salivarius* LI01 alleviated OVX‐induced osteoporosis in mice. Furthermore, LI01 enhanced the de novo synthesis of GSH by increasing the levels of its key enzyme Gclc, and the increased GSH inhibited CREB activation, resulting in suppressed mitochondrial biogenesis and reduced mitochondrial ROS production.

## CONFLICT OF INTEREST

All authors have no conflict of interests.

## AUTHOR CONTRIBUTIONS

YY and SN designed and performed this study and drafted the manuscript. AXZG and JY analysed the data. SN revised the paper. All authors read and approved the final manuscript.

## Supporting information

Fig S1

Fig S2

Fig S3

Table S1

## Data Availability

All data sets used and/or analysed during the current study are available from the corresponding author on reasonable request.
