# Supplementary figures and images for: Gut microbiota modulates osteoclast glutathione synthesis and mitochondrial biogenesis in mice subjected to ovariectomy
Source: Cell Prolif. 2022 Jan 26;55(3):e13194. doi: 10.1111/cpr.13194 (PMC8891549; doi:10.1111/cpr.13194)

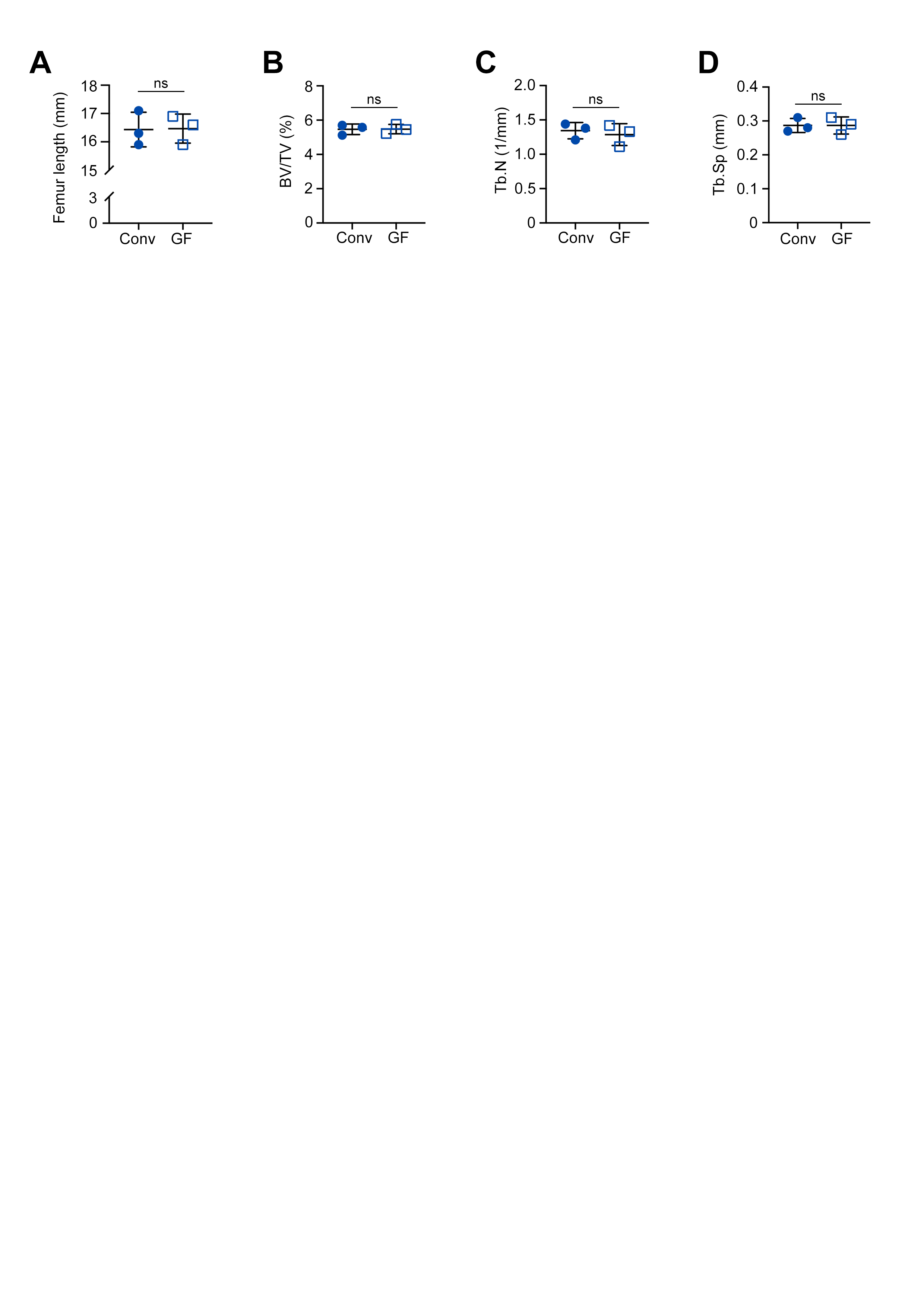

Supplement: Supplementary file 1 — Fig S1 [file CPR-55-e13194-s002.tif]

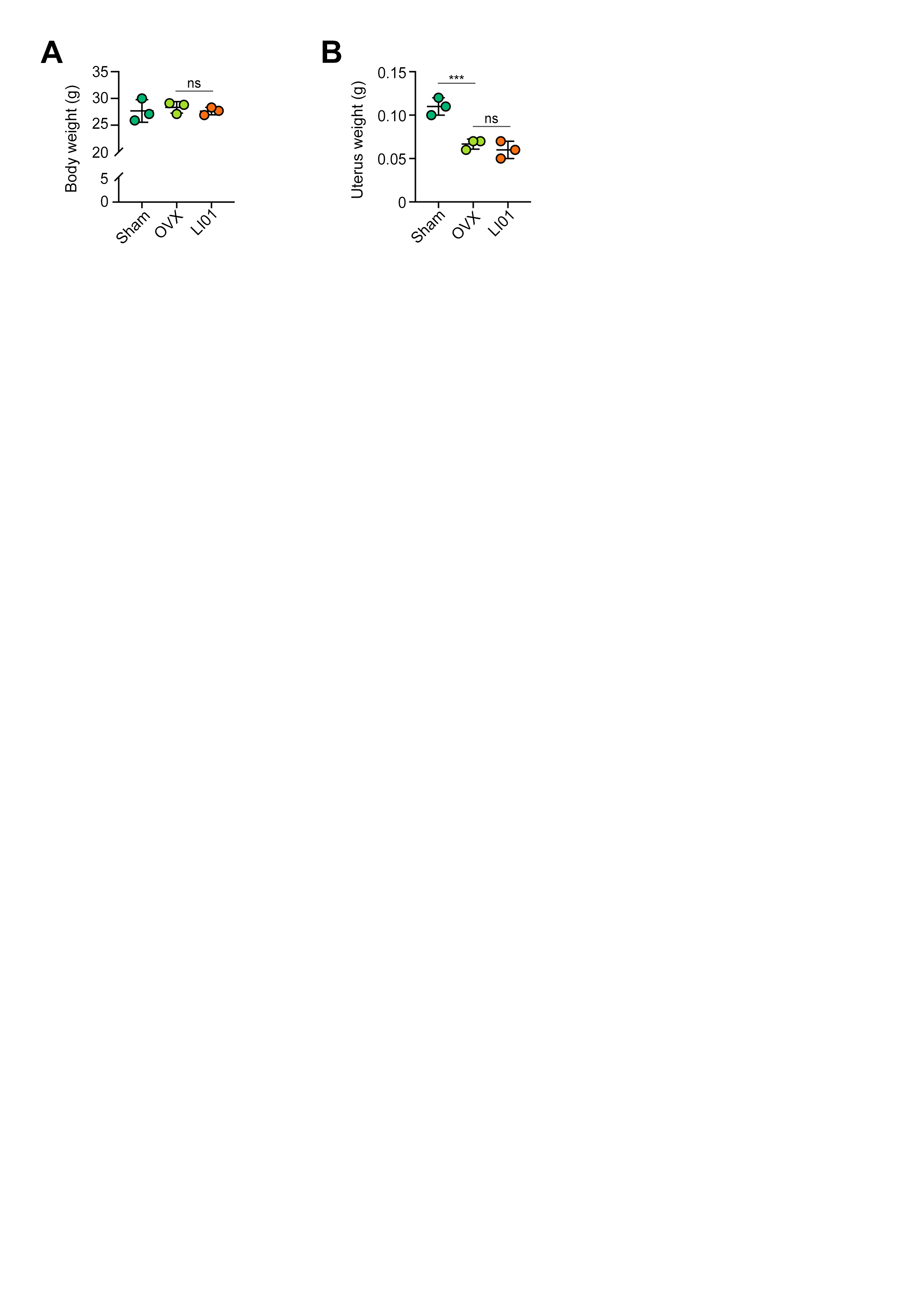

Supplement: Supplementary file 2 — Fig S2 [file CPR-55-e13194-s003.tif]

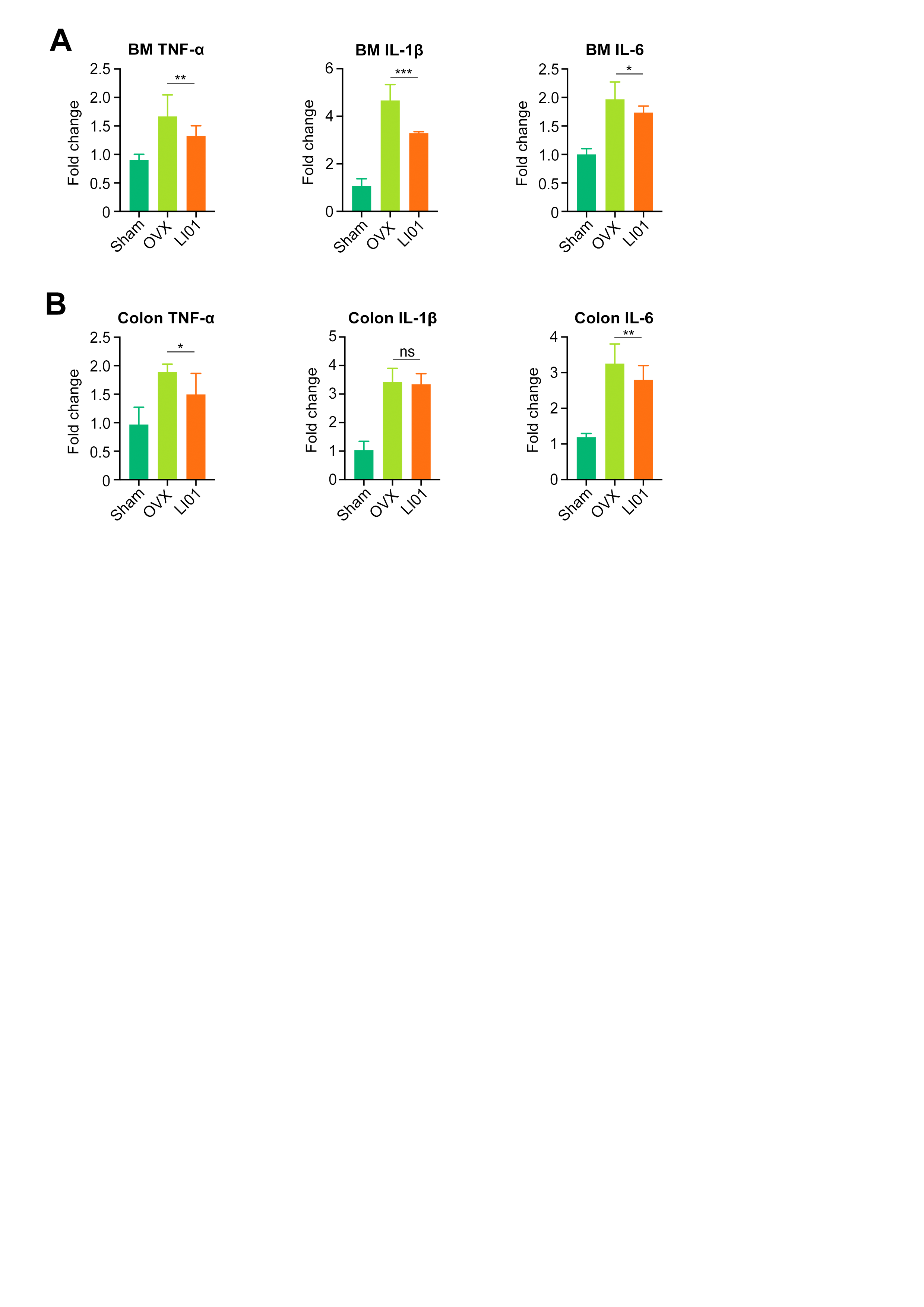

Supplement: Supplementary file 3 — Fig S3 [file CPR-55-e13194-s001.tif]
