# Supplementary material for: Gut microbiota modulates osteoclast glutathione synthesis and mitochondrial biogenesis in mice subjected to ovariectomy
Source: Cell Prolif. 2022 Jan 26;55(3):e13194. doi: 10.1111/cpr.13194 (PMC8891549; doi:10.1111/cpr.13194)
Supplement: Supplementary file 4 — Table S1 [file CPR-55-e13194-s004.docx]

Supplementary Table 1. Primers for qRT-PCR

| **Genes** | **Forward (5′-3′)** | **Reverse (5′-3′)** |
| --- | --- | --- |
| ***OCN*** | GAGGGCAATAAGGTAGTGA ACAGA | AAGCCATACTGGTTTGATAGCTCG |
| ***RUNX-2*** | TTCTCCAACCCACGAATGCAC | CAGGTACGTGTGGTAGTGAG |
| ***ALP*** | ACCACCACGAGAGTGAACCA | CGTTGTCTGAGTACCAGTCCC |
| ***TRAP*** | AGTAAGGGCTGGGAAGT | AGCGACAAGAGGTTCCAG |
| ***CTSK*** | CTTCCAATACGTGCAGCAGA | TCTTCAGGGCTTTCTCGTTC |
| ***c-FOS*** | GCGAGCAACTGAGAAGAC | TTGAAACCCGAGAACATC |
| ***MMP9*** | CGTGTCTGGAGATTCGACTTGA | TTGGAAACTCACACGCCAGA |
| ***NFATc1*** | CAACGCCCTGACCACCGATAG | GGCTGCCTTCCGTCTCATAGT |
| ***Gclc*** | AACAAGAAACATCCGGCATC | CGTAGCCTCGGTAAAATGGA |
| ***Gclm*** | TGGAGCAGCTGTATCAGTGG | CCTTTTGGCTTGCAGAATGT |
| ***GSS*** | GTACTCACTGGATGTGGGTGAAGA | CGGCTCGATCTTGTCCATCAG |
| ***GSR*** | CTTTCAGCTGGAGGACTTGC | CCAGGCCTGATGATGTCTTT |
| ***NFE2L2*** | TCTCCTCGCTGGAAAAAGAA | AATGTGCTGGCTGTGCTTTA |
| ***Keap1*** | AGATCGGCTGCACTGAACTG | GGCAGTGTGACAGGTTGAAG |
| ***TNF-α*** | CATCTTCTCAAAATTCGAGTGACAA | TGGGAGTAGACAAGGTACAACCC |
| ***IL-6*** | GAGGATACCACTCCCAACAGACC | AAGTGCATCATCGTTGTTCATACA |
| ***IL-1β*** | CAACCAACAAGTGATATTCTCCATG | GATCCACACTCTCCAGCTGCA |
| ***CYTB*** | GGGTTCTATTGTTTTTTTGCCT | ACCGATCTACTACCAGTATAAT |
| ***COX2*** | AACCGAGTCGTTCTGCCAAT | CTAGGGAGGGGACTGCTCAT |
| ***ND1*** | GCAGCAACGTATTGGTCCTA | AAACAAAGCATAAGAAACAC |
| ***GAPDH*** | ATCATCCCTGCATCCACT | TCTTCAGGGCTTTCTCGTTC |
